# Editorial: Day-care for healthy child development and wider social and economic gain in urban areas in low- and middle income countries

**DOI:** 10.3389/fpubh.2024.1386958

**Published:** 2024-03-25

**Authors:** Margaret Nampijja, Patricia Kitsao-Wekulo, Robert C. Hughes, Paula Griffiths, Helen Elsey

**Affiliations:** ^1^African Population and Health Research Center, APHRC Campus, Nairobi, Kenya; ^2^Department of Population Health, Faculty of Epidemiology and Population Health, London School of Hygiene and Tropical Medicine, London, United Kingdom; ^3^School of Sport, Exercise and Health Sciences, Loughborough University, Loughborough, United Kingdom; ^4^Department of Health Sciences, University of York, York, United Kingdom

**Keywords:** daycare, low income and middle income countries, cities, child development, nurturing care framework (NCF)

## Background

Rapid urbanization and socio-economic change in low- and middle-income countries (LMICs) has triggered a childcare crisis (1). As families move to the city, they leave behind traditional support networks; parents, particularly mothers, are working long hours outside the home often in informal and unstable jobs. In response to the increasing demand, informal private day cares have sprung up. Governments' responses to the pressure have been varied, and due to the intersectoral nature of early childhood development (ECD), the care for young children has often fallen between ministries (1, 2). These factors have left children from low-income families with substandard childcare (3) and at high risk of poor health and development.

This Research Topic brings the global challenge of the childcare vacuum to the attention of academics and policy makers from multiple disciplines. Despite the growing need across low and middle income countries (LMICs) to better understand childcare within low-income families, urban settings, and other complex environments, and which responses are most appropriate, the evidence base is limited (4). Systematic reviews repeatedly find limited studies of effective center-based childcare interventions to improve ECD and health in LMICs (5–7). Evidence from both high income countries (HICs) (8) and LMICs (9) indicates that cheap, but poor quality center-based child-care may worsen ECD outcomes. Cognizant of the trans-disciplinary approach to this global challenge, the special issue brings together a range of papers to explore the different dimensions of center-based childcare including the demand, policy implications, and childcare models that can feasibly, sustainably and effectively be delivered in low-income and complex urban-poor neighborhoods in the global south.

Global evidence highlights the need for a multifaceted approach to day-care centers addressing hygiene, nutrition, safety and nurturing responsive, emotionally supportive and developmentally enriching relationship between the child and caregiver (10) and this has formed the basis of the WHO's Nurturing Care Framework (NCF) (11). The NCF is a broad framework for supporting the development of children aged 0–3years, which recommends that environments where children live should be healthy, safe, hygienic, and provide nutritious food and responsive nurturing care. None of the components are sufficient on their own, and instead, work together synergistically to promote healthy childhood development. The period below 3 years is critical for a child's development since adverse exposures during this period can have lasting implications for their development and future success as adults (12). Key themes are highlighted in this editorial, and discussed in detail in the individual publications.

## Nutrition

Despite the sustainable development goals (SDGs) targets, WHO's nurturing care guidelines, and government efforts in ensuring optimum nutrition and growth for all young children, worldwide there is still a high burden of malnutrition, with an estimated 45.4 million children under age 5 wasted and 149.2 million stunted (13). Several factors, including poor caregiver knowledge and practices of infant and child feeding, explain the persistently high rate of malnutrition especially in low income settings (14, 15). Amoah et al.'s paper published in this Research Topic examines minimum dietary requirements as a driver of malnutrition among children in an urban-poor setting in Ghana. The research revealed a significant gap in dietary diversity, with less than half of the children below 2 years meeting minimum dietary diversity (MDD) requirements, and poor caregiver knowledge and practices of infant and young child feeding identified as key contributors to poor MDD. Similar nutrition gaps were found in informal day-care centers in Nairobi (Nampijja, Langat, Oloo, Okelo et al.). Clearly, interventions including educating caregivers on infant and young child feeding are needed to ensure optimum nutrition for all children.

## Safety and responsive care

Young children living in extreme levels of poverty found in informal settlements, as well as in institutionalized care are prone to receiving inadequate care and nurturing across the NCF domains. As revealed by Onayemi and Hapunda's study which is one of the few conducted in orphanages in Nigeria. Multiple individual, institutional and policy level challenges constrain provision of quality childcare in these homes. These include weak bonding between carers and the children, stigma and un-responsive caregiver attitudes. Building the evidence base to understand how care in these settings can be improved to counter the negative impacts on the socioemotional wellbeing of children is needed. The paper highlights how the lack of funds including from governments, and poor implementation of policies undermines the ability to meet the health, nutrition and development needs of the children threatening their safety and wellbeing. In Kenya, safe and responsive care issues were reported in “baby cares” in an informal settlement, and lack of resources, knowledge and skills among care providers as well as inadequate communication between parents and caregivers were major barriers to quality childcare provision (Jegathesan et al.). Cost-effective and sustainable policy and programming interventions are critical for raising the standards of childcare services in these environments for holistic child growth and development.

## WASH

Poor water, sanitation and hygiene (WASH) services in childcare centers in low income settings put children at high risk for diarrhea and other diseases (5). Poor infrastructure and limited government resources underlie poor WASH services in these communities, yet formal social accountability mechanisms to seek improvement from local government, public or private providers have often not been successful. Chumo et al.'s paper presents a novel approach to improving hygiene and sanitation within resource constrained environments. The study demonstrates that an informal social accountability process codesigned with the community and focusing on defining the issues, identifying actions, sharing information, learning and adaptation has the potential to increase the capability of even poorly resourced childcare centers to better meet sanitation and hygiene needs in childcare centers. While this does not absolve formal providers and local governments of their responsibility to provide equitable WASH service delivery, it is clear that programs are more appropriate and sustainable when communities are fully engaged in co-creation of solutions to problems that affect them.

## Policy

Given the diverse challenges that affect childcare, it is useful to understand the policy landscape across the domains of the NCF. A comprehensive review of ECD policies and plans in Kenya conducted by Abboah-Offei et al. highlights key limitations including in responsive care, early learning, safety and security, and the role of fathers role in childcare. Owino and Yigezu explore fathers' involvement in childcare in urban informal settlements and those attached to commercial farms in Kenya and Ethiopia. While there are interesting differences between the two countries, the overarching similarity is that fathers still play a predominantly financial role in the care of their children. The other elements of the NCF are mainly delivered by mothers and other female caregivers. Interestingly, in both countries, use of childcare centers was relatively low, but other forms of paid childcare including use of housemaids occurred. In a few cases, children were left under the care of older siblings or neighbors, most likely because of the inability to afford paid childcare. Governments should invest in childcare services to enable low income families to access quality affordable childcare for their children. Owino and Yigezu's paper highlights the diminishing traditional family support structures, rapidly evolving gendered work patterns, and the resulting shifts in caring roles, as an important focus for research and policy consideration.

## Interventions

Interventions that respond to one or more issues raised above are needed to optimize care for young children in the urban poor communities. Three papers from one study done in Kenya (16) highlight that interventions should be designed to fit a given context, to address the existing knowledge gaps, and maximize stakeholder engagement in their conceptualization and implementation. Initiatives that leverage local (government) structures for integration, sustainability and scaling are more likely to be successful as presented in the three publications. First, a survey of childcare centers examined the quality and key drivers of quality in childcare centers in the Nairobi slums, with poor caregiver knowledge and practices being the major barrier (Nampijja, Langat, Oloo, Okelo et al.). The second paper describes a successful codesign process with center owners, NGOs, and lay community members to develop a community of practice approach to improving day-care center provider skills and practice (17). In the third paper, with implementation led by government-supported community health volunteers (CHVs) and supervised by community health assistants (CHAs), with training from an NGO with expertise in childcare (Kidogo), the intervention was found to be feasible and showed potential to improve the knowledge and practices of the center providers (Nampijja, Langat, Oloo, Amboka et al.). Hence initiatives that promote joint stakeholder participation and ownership, and which are embedded in government structures but also align with policies have a high chance of thriving. Government's role in providing guidance and support, policy framework and supervision through community health and ECD teams is important for projects and sustainability beyond the project life. We now also need more studies which track changes in developmental outcomes in relation to the improvement in the different spheres of day-care.

## Conclusion

Overall, the issues and opportunities in childcare spotlighted in this Research Topic span the entire NCF, at both policy and practice levels. Governments' support and coordination with other actors is paramount. Children living in socioeconomic disadvantage deserve special priority given their unique vulnerabilities. In the context of limited resources, low cost co-created and integrated approaches are key for sustainability. A good understanding of the specific barriers in complex low-income urban neighborhoods, and impact of specific solutions is vital for informing future investment in childcare. Lastly, facilitated shared learnings within and between countries are crucial for promoting best practices in a wider positive community of practice (18).

## Author contributions

MN: Conceptualization, Writing—original draft, Writing—review & editing. PK-W: Writing—review & editing. RH: Writing—review & editing. PG: Writing—review & editing. HE: Conceptualization, Writing—original draft, Writing—review & editing.
